# Body mass index (BMI) and forensic autopsy: association with procedural duration, technical complexity and operator vigilance

**DOI:** 10.1007/s00414-026-03859-1

**Published:** 2026-06-05

**Authors:** Isabella Aquila, Matteo Antonio Sacco

**Affiliations:** Institute of Legal Medicine, Department of Medical and Surgical Sciences, “Magna Graecia” University, Catanzaro, 88100 Italy

**Keywords:** Body mass index, Obesity, Forensic pathology, Autopsy duration, Vigilance decrement, Human factors

## Abstract

Body mass index (BMI) is routinely recorded in forensic autopsy practice and represents a well-established risk factor for numerous natural causes of death. However, its role as a procedural modifier influencing autopsy workflow, technical complexity, and operator cognitive performance has not been systematically investigated. This study aimed to evaluate the impact of BMI on autopsy duration, anatomical–technical difficulty, and operator vigilance under standardized medico-legal conditions.

A total of 50 consecutive medico-legal autopsies performed for natural deaths were prospectively analyzed, including an obese cohort (*n* = 25, BMI ≥ 30 kg/m²) and a non-obese control group (*n* = 25, BMI < 30 kg/m²), matched for age and sex and examined using an identical standardized autopsy protocol. The term “consecutive” refers to all eligible medico-legal autopsies for natural deaths performed during the study period, excluding cases of violent or suspicious death and those not meeting inclusion criteria. The cases were collected over a period of approximately six months, during which all qualifying autopsies were prospectively included. Body mass index was analyzed both as a continuous variable and according to obesity severity classes. Autopsy duration was objectively measured from the first skin incision to completion of organ removal. Operator vigilance was assessed using the Mackworth Clock Test, administered to both primary and secondary operators immediately before and after each autopsy. Morphometric assessment of subcutaneous adipose tissue thickness at the thoracoabdominal incision site and qualitative evaluation of technical difficulty were also performed.

Autopsy duration was significantly longer in obese cases compared with controls (mean 219.6 ± 26.9 vs. 184.2 ± 14.9 min; *p* < 0.001), with a progressive increase across obesity classes. Within the obese cohort, BMI was a significant predictor of procedural duration, with an estimated increase of approximately 4 min per BMI unit. *Increased autopsy duration was associated with an exploratory signal of vigilance decrement*,* which was greater in obese cases than in controls*. Each additional 30 min of procedural time was associated with an increase of approximately one missed critical stimulus on post-autopsy vigilance testing. In all cases with BMI ≥ 35 kg/m², subcutaneous adipose tissue thickness exceeded 4 cm, correlating with deeper incision planes, delayed cavity access, and increased technical difficulty.

These findings suggest that obesity may act as a procedural modifier in forensic autopsy practice and support further investigation of workload-related cognitive effects under standardized conditions.

## Introduction

Body mass index (BMI) represents one of the most extensively validated anthropometric parameters for the classification of overweight and obesity and is widely employed in clinical, epidemiological, and public health research [1]. Defined as the ratio between body weight and the square of height, BMI provides a standardized and reproducible surrogate of overall adiposity that enables interindividual and population-level comparisons. Despite its limitations, BMI remains the reference metric for stratifying obesity-related risk [1, 2]. In clinical medicine, elevated BMI has been consistently associated with increased incidence of cardiovascular disease, metabolic syndrome, type 2 diabetes, thromboembolic events, and sudden cardiac death, while in forensic pathology a growing body of autopsy-based literature has confirmed its correlation with structural cardiac alterations, coronary atherosclerosis, epicardial adipose tissue expansion, pulmonary thromboembolism, and obesity-related cardiomyopathy [1–3]. Despite this consolidated pathogenic relevance, BMI is still largely treated in routine forensic practice as a descriptive variable rather than as an active determinant capable of influencing the autopsy process itself.

From an anatomical and procedural standpoint, obesity profoundly modifies the internal landscape encountered during autopsy. Obesity may reduce the visibility of anatomical structures and complicate in situ evaluation due to excessive visceral and subcutaneous adipose tissue. These modifications are particularly evident in the thoracoabdominal compartment, where adipose infiltration affects not only the abdominal wall but also the mesentery, retroperitoneum, and perivisceral spaces, thereby complicating organ exposure, mobilization, and removal. In addition to the thoracoabdominal and retroperitoneal regions, the cervical district represents a critical area in forensic investigations. In obese subjects, increased adipose tissue in the neck may obscure anatomical landmarks and complicate dissection. Autopsy-based morphometric studies have further demonstrated that increased BMI correlates with increased thickness of subcutaneous adipose tissue, augmented epicardial and perivascular fat, and greater visceral adiposity, all of which contribute to increased resistance during dissection and prolongation of operative steps [1–3]. In this context, BMI may be conceptualized as a biomechanical modifier that directly impacts the physical workload required to complete a forensic autopsy.

Despite this expanding evidence, the procedural consequences of elevated BMI on autopsy workflow remain largely unexplored in the forensic literature. In particular, no systematic investigation has yet addressed whether BMI influences autopsy duration, technical execution, or the cognitive performance of operators engaged in prolonged dissections. This gap is particularly relevant when considering that forensic autopsies are complex, physically demanding procedures that require sustained attention, fine motor control, and continuous situational awareness over extended periods of time. Forensic autopsies require sustained attention and procedural precision throughout the examination [4–9].

Human-factors research has shown that prolonged and demanding tasks may contribute to fatigue and vigilance decline [10–12]. The Mackworth Clock Test is a validated tool for assessing sustained attention, although its application in forensic pathology has been limited. In the context of obesity, it is reasonable to hypothesize that increased procedural duration and technical complexity may exacerbate cognitive fatigue and reduce vigilance. Longer dissections may increase procedural workload and operator fatigue during autopsy performance. The present study was therefore designed to investigate BMI not merely as a pathological correlate but as an independent procedural and cognitive modifier within forensic autopsy practice. By integrating objective measurements of autopsy duration, standardized assessment of operator vigilance, and qualitative evaluation of technical difficulty, this experimental investigation aims to provide *a* systematic analysis of how BMI influences the dynamics of medico-legal autopsy execution.

## Materials and methods

### Study design and conceptual framework

**Fig. 1:**
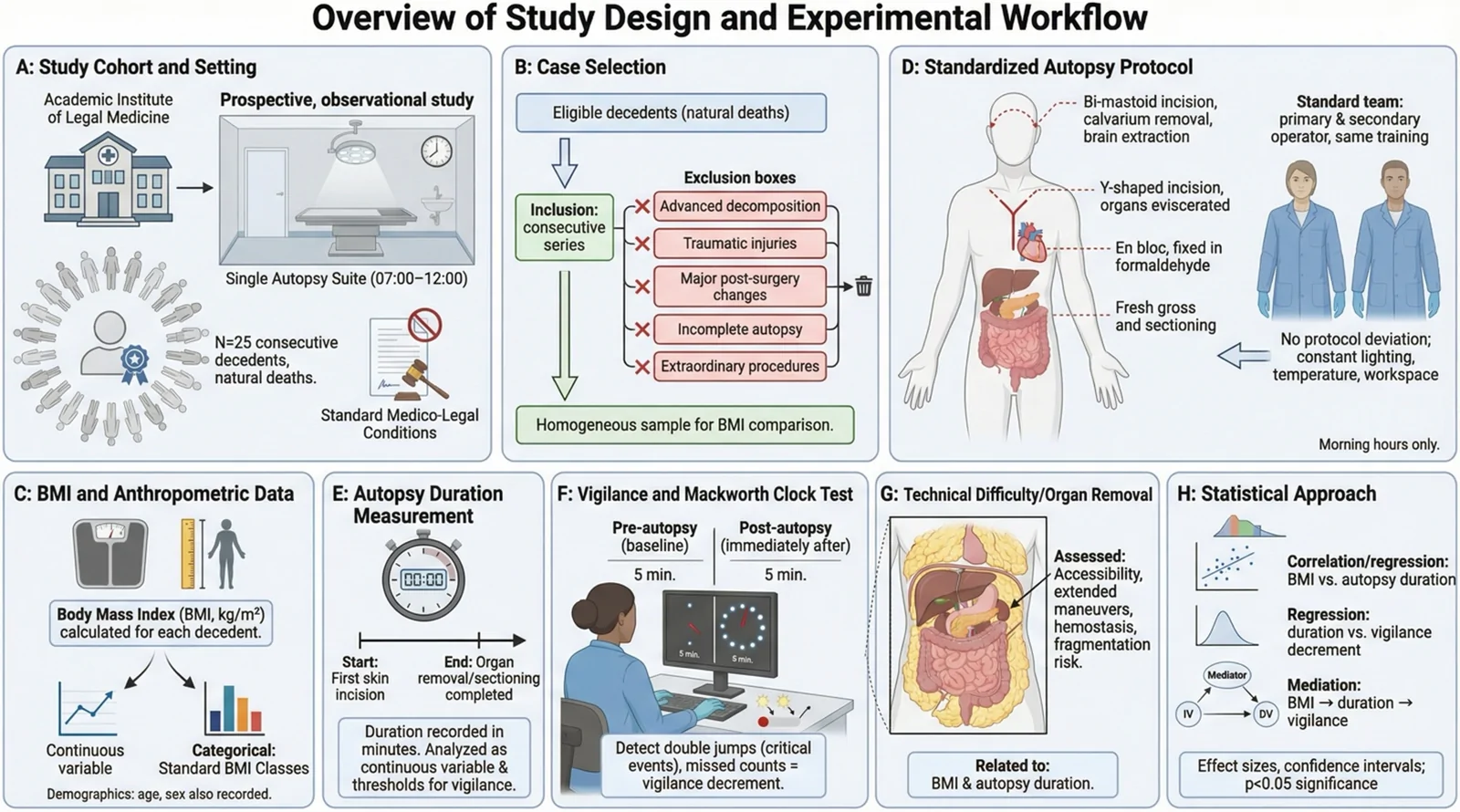
Overview of study design and experimental workflow

This study was designed as a prospective observational investigation aimed at characterizing the procedural and cognitive impact of obesity on forensic autopsy practice under standardized medico-legal conditions. The conceptual framework integrates three complementary dimensions: procedural workload, quantified through objective measurement of autopsy duration; cognitive performance, assessed via validated vigilance testing; and anatomical–technical complexity, evaluated through morphometric and qualitative observations (Fig. 1).

The figure summarizes the prospective observational design, case selection criteria, standardized autopsy protocol, anthropometric assessment, objective measurement of autopsy duration, operator vigilance testing using the Mackworth Clock Test, evaluation of technical difficulty, and the statistical framework adopted to investigate BMI-related procedural and cognitive effects under controlled medico-legal conditions.

### Case selection, eligibility criteria, and grouping

A total of 50 consecutive medico-legal autopsies performed for natural deaths at Institute of Legal Medicine of Magna Graecia University of Catanzaro were prospectively included. All participating operators provided informed consent for inclusion in the study. The study population was divided into two groups based on BMI: an obese cohort (*n* = 25) and a non-obese control group (*n* = 25). Inclusion in the obese cohort required a BMI ≥ 30 kg/m², while controls were defined by a BMI < 30 kg/m². The control group was selected to be comparable to the obese cohort with respect to age and sex distribution, in order to minimize demographic confounding. Exclusion criteria for both groups included advanced decomposition, extensive traumatic injuries, major post-surgical anatomical alterations, incomplete autopsy examinations, or the need for extraordinary investigative procedures that could independently prolong autopsy duration. Extraordinary procedures were defined as additional investigative steps beyond routine autopsy practice, including extensive toxicological sampling, radiological imaging (e.g., post-mortem CT), complex reconstruction procedures, or multidisciplinary interventions that could significantly prolong the examination time. Major post-surgical anatomical alterations included extensive prior surgical resections, anatomical reconstructions, or conditions significantly modifying normal organ relationships. Cases involving organ donation were also considered within this category and were therefore excluded. Although the specific causes of death were not completely uniform across cases, the study design minimized major procedural heterogeneity by excluding traumatic deaths, advanced decomposition, massive generalized edema, extensive surgical alterations, and other conditions potentially associated with unusually prolonged examinations. In addition, no evident imbalance in the distribution of major natural cause-of-death categories was observed between obese and control groups, reducing the likelihood that the observed differences in procedural duration were primarily driven by pathological variability rather than BMI-related anatomical complexity.

### Anthropometric data collection and BMI classification

Body weight and height were directly measured in all cases prior to autopsy using standard mortuary procedures. When available, these measurements were cross-checked with recent antemortem clinical records. This approach was adopted to ensure accuracy in BMI estimation, taking into account potential post-mortem changes such as dehydration, which may affect body weight. [11, 12]. BMI was treated as both a continuous variable and a categorical variable in subsequent analyses. Within the obese cohort, cases were stratified into obesity class I (BMI 30.0–34.9 kg/m²), class II (BMI 35.0–39.9 kg/m²), and class III (BMI ≥ 40.0 kg/m²) according to internationally accepted thresholds, allowing exploration of dose–response relationships across increasing obesity severity. BMI classification was performed according to World Health Organization (WHO) guidelines [13–17].

### Autopsy team composition and operator standardization

All autopsies were performed by the same fixed team consisting of a primary operator and a secondary operator, both experienced forensic pathologists with comparable training and years of practice. Maintaining a constant team composition was a deliberate methodological choice to minimize inter-operator variability in technical skill, speed, and fatigue susceptibility. The roles of primary and secondary operator were kept consistent across all cases, with the primary operator performing the core dissection and organ removal steps and the secondary operator assisting and documenting findings.

### Procedural conditions and environmental control

All examinations were conducted in the same autopsy suite during routine institutional activity from Monday to Friday, between 07:00 and 12:00. No autopsies included in the study were performed during night shifts or public holidays. This time window was selected to reduce circadian influences on attention, alertness, and fatigue, which are known to affect cognitive performance. Although the specific weekday was not analyzed as an independent variable, the distribution of cases across the regular working week reduced the likelihood of systematic bias related to accumulated operator fatigue. Environmental parameters such as lighting, room temperature, and workspace organization were maintained as constant as possible throughout the study period. Both obese cases and controls were examined under identical procedural and environmental conditions, ensuring that differences in duration or vigilance were not attributable to extrinsic factors.

### Standardized autopsy protocol

A uniform autopsy protocol was strictly applied to all cases. Cranial access was achieved through a bi-mastoid incision with removal of the calvarium and routine brain extraction. Thoracic and abdominal cavities were opened using a Y-shaped incision. Complete evisceration of thoracic and abdominal organs was performed in all cases, followed by fresh gross examination and systematic sectioning of all organs. The heart was consistently removed en bloc and subjected to systematic fresh dissection during the autopsy, followed by fixation in formaldehyde for subsequent detailed examination. Also, the remaining organs were examined fresh according to institutional routine practice. No deviations from this protocol were permitted, and no procedural shortcuts were allowed, even in technically challenging cases, in order to preserve methodological comparability.

### Measurement and operationalization of autopsy duration

Autopsy duration was measured objectively in minutes using a standardized timing protocol. The start time was defined as the moment of the first skin incision, and the end time corresponded to completion of organ removal and sectioning, immediately prior to reconstruction of the body. Autopsy duration was analyzed as a continuous variable and additionally categorized into duration intervals to explore nonlinear associations with vigilance decrement.

### Vigilance assessment and Mackworth Clock Test protocol

Operator vigilance was assessed using the Mackworth Clock Test, a validated and widely used paradigm for evaluating sustained attention and vigilance decrement during prolonged tasks. The test was administered individually to both the primary and secondary operators immediately before the start of each autopsy and immediately after its completion. Each testing session lasted five minutes and involved continuous monitoring of a rotating clock hand, during which infrequent critical events, operationally defined as double jumps of the second hand, occurred at irregular intervals. Operators were instructed to respond to each critical event via a standardized input mechanism. The number of missed critical stimuli was recorded as the primary vigilance outcome. Vigilance decrement was operationalized as the difference between post-autopsy and pre-autopsy test performance, allowing within-subject control for baseline attentional variability.

### Morphometric assessment of adipose tissue and technical observations

Morphometric assessment of subcutaneous adipose tissue thickness was performed at the thoracoabdominal incision site during each autopsy, providing an objective anatomical parameter related to dissection difficulty [11, 12]. Particular attention was paid to cases with higher BMI values. In parallel, technical difficulty was qualitatively documented throughout the procedure, focusing on exposure, mobilization, and removal of anatomically deep or adipose-surrounded organs, including the liver, pancreas, adrenal glands, and pelvic organs. Technical observations included the need for extended incision depth, delayed access to body cavities, prolonged mobilization maneuvers, and increased manipulation of perivisceral adipose tissue.

### Statistical analysis and modeling strategy

Descriptive statistics were used to summarize demographic, anthropometric, procedural, and vigilance-related variables, expressed as mean ± standard deviation and range as appropriate. Distributional properties were assessed prior to inferential analysis to guide the choice of parametric or non-parametric methods. Between-group comparisons between obese cases and controls were conducted using appropriate statistical tests based on data distribution. Associations between BMI and autopsy duration were explored using correlation analysis and linear regression modeling, with BMI entered as a continuous predictor. Regression models were adjusted for age and sex to assess potential confounding.

To evaluate the relationship between procedural workload and cognitive performance, vigilance decrement was modeled as a function of autopsy duration using linear regression. An indirect-effect analytical framework was applied to assess whether the effect of BMI on vigilance decrement was mediated by procedural duration. Given the exploratory nature of the vigilance-related analyses and the moderate sample size, findings should be interpreted cautiously and considered hypothesis-generating. All analyses were conducted in accordance with accepted biostatistical standards for exploratory forensic and human-factors research.

## Results

A total of 50 medico-legal autopsies performed for natural deaths were included, comprising an obese cohort (*n* = 25) and a non-obese control group (*n* = 25), examined under identical procedural conditions and applying the same standardized autopsy protocol. The obese cohort included 14 males and 11 females, with a mean age of 67.4 ± 11.2 years, while the control group included 14 males and 11 females, with a mean age of 66.9 ± 10.8 years, indicating close demographic comparability. Body mass index in the obese cohort ranged from 30.2 to 44.6 kg/m² (mean 36.8 ± 4.1 kg/m²), whereas controls ranged from 24.0 to 29.8 kg/m² (mean 27.1 ± 1.6 kg/m²). Within the obese cohort, 9 cases were classified as obesity class I (BMI 30.0–34.9), 8 as class II (BMI 35.0–39.9), and 8 as class III (BMI ≥ 40.0), enabling intra-obesity gradient analyses [9, 10].

Total autopsy duration showed a marked between-group difference. In the obese cohort, duration ranged from 188 to 258 min (mean 219.6 ± 26.9), whereas in controls it ranged from 160 to 210 min (mean 184.2 ± 14.9). The difference in mean duration between obese cases and controls was statistically significant (mean difference 35.4 min; 95% CI: 23.1–47.7; *p* < 0.001). When the obese cohort was stratified by severity class, a progressive increase in duration was observed (class I: 198.4 ± 14.7; class II: 222.9 ± 16.8; class III: 246.1 ± 12.9 min), with a statistically significant trend (*p* < 0.001) and minimal overlap between class I and class III.

When BMI was analyzed as a continuous predictor, significant positive associations with procedural duration were confirmed both within the obese cohort and across the full sample. Within the obese cohort, Pearson correlation between BMI and autopsy duration was *r* = 0.62 (*p* = 0.001), and linear regression estimated an increase of 4.3 min per BMI unit (β = 4.3, 95% CI: 2.4–6.2; *p* = 0.001). Across the entire sample (obese + controls), the BMI–duration association remained significant (*r* = 0.71, *p* < 0.001), and in a multivariable regression model including age and sex, BMI remained an independent predictor of duration, with no material effect modification by sex and no significant confounding by age. A model including group (obese vs. control) as a categorical predictor confirmed a significant obesity-related increase in autopsy duration even after accounting for BMI as a continuous variable.

Baseline Mackworth Clock Test performance was comparable between groups, indicating similar pre-autopsy vigilance. For the primary operator, baseline missed stimuli averaged 1.2 ± 0.6 in obese cases and 1.1 ± 0.6 in controls. Post-autopsy testing showed a duration-dependent vigilance decrement in both groups, but markedly greater in obese cases. The primary operator’s vigilance decrement (Δ missed stimuli, post minus pre) averaged 4.4 ± 1.2 in obese cases versus 2.4 ± 0.9 in controls (mean difference 2.0; 95% CI: 1.4–2.6; *p* < 0.001). In the obese cohort, decrement increased with obesity severity, with the highest post-autopsy error burden observed in class III cases, paralleling longer procedural duration. Regression analysis identified an exploratory association between longer procedural duration and increased missed stimuli in the primary operator (β = 1.10, 95% CI: 0.52–1.68;p = 0.002). Secondary operator trends were similar but attenuated (Tables 1, 2, 3, 4 and 5).

**Table 1:** Demographic and anthropometric characteristics (obese cohort vs. controls)

Variable	Obese cohort (*n* = 25)	Controls (*n* = 25)
Sex (male/female)	14 / 11	14 / 11
Age, mean ± SD (years)	67.4 ± 11.2	66.9 ± 10.8
BMI, mean ± SD (kg/m²)	36.8 ± 4.1	27.1 ± 1.6
BMI range (kg/m²)	30.2–44.6	24.0–29.8
Obesity class I (30.0–34.9), n	9	—
Obesity class II (35.0–39.9), n	8	—
Obesity class III (≥ 40.0), n	8	—

**Table 2:** Autopsy duration (obese cohort vs. controls)

Group	*n*	Autopsy duration, mean ± SD (min)	Range (min)
Controls (BMI < 30)	25	184.2 ± 14.9	160–210
Obese cohort (BMI ≥ 30)	25	219.6 ± 26.9	188–258

**Table 3:** Autopsy duration by obesity class (within obese cohort)

Obesity class	*n*	Autopsy duration, mean ± SD (min)	Range (min)
Class I (30.0–34.9)	9	198.4 ± 14.7	188–215
Class II (35.0–39.9)	8	222.9 ± 16.8	205–240
Class III (≥ 40.0)	8	246.1 ± 12.9	230–258

**Table 4:** Mackworth Clock Test: vigilance decrement (primary operator) in obese vs. controls

Group	Baseline missed stimuli (mean ± SD)	Post-autopsy missed stimuli (mean ± SD)	Δ missed stimuli (mean ± SD)
Controls (*n* = 25)	1.1 ± 0.6	3.5 ± 0.9	2.4 ± 0.9
Obese (*n* = 25)	1.2 ± 0.6	5.6 ± 1.1	4.4 ± 1.2

**Table 5:** Key associations: BMI, autopsy duration, and vigilance

Analysis	Estimate	95% CI	*p*-value
BMI vs. autopsy duration (obese cohort), Pearson r	0.62	0.30–0.81	0.001
BMI → duration (obese cohort), β (min/BMI unit)	4.3	2.4–6.2	0.001
BMI vs. autopsy duration (full sample), Pearson r	0.71	0.53–0.83	< 0.001
Duration → vigilance decrement, β (Δ per + 30 min)	1.10	0.52–1.68	0.002

Exploratory analyses suggested that the association between BMI and vigilance decrement was largely related to procedural duration. BMI was significantly associated with autopsy duration, and autopsy duration was associated with vigilance decrement. When duration was included in models predicting vigilance change, attenuation of the direct BMI term was observed. These findings should be interpreted cautiously given the exploratory design and the use of a fixed operator team.

Morphometric and technical observations further corroborated the quantitative findings. In all cases with BMI ≥ 35 kg/m² (obesity classes II–III), the thickness of the subcutaneous adipose tissue at the thoracoabdominal incision site exceeded 4 cm, whereas in controls adipose thickness remained consistently below 4 cm. Increased adipose thickness was associated with deeper incision planes, delayed entry into the cavities, and prolonged dissection times. Organ-specific technical difficulty was most pronounced in obese class II–III cases during exposure and mobilization of liver, pancreas, adrenal glands, and deep pelvic organs, where abundant perivisceral fat and altered anatomical relationships required extended dissection and increased manipulation, closely paralleling increases in duration and vigilance decrement.

The relatively long average duration observed in both groups likely reflects the strict standardization of the protocol and the inclusion of additional procedural assessments.

## Discussion

The present study provides a comprehensive experimental evaluation of the impact of obesity on forensic autopsy workflow, integrating procedural, anatomical, and cognitive dimensions under a standardized medico-legal setting. The observed prolongation of autopsy duration in obese decedents, together with the graded increase across obesity severity classes, confirms that obesity exerts a dose-dependent effect on the technical execution of post-mortem examinations, even when all other procedural variables are tightly controlled [18–20].

The difference in mean procedural time between obese and control cases, coupled with the persistence of a significant BMI–duration association across the full sample, supports the conclusion that once BMI reaches the obese range, the autopsy workflow undergoes a qualitative shift toward increased complexity and workload [21–23]. This finding is particularly relevant in contemporary forensic practice, given the rising prevalence of obesity in the general population and the consequent increase in obese decedents undergoing medico-legal examination.

A key contribution of the present study lies in the integration of cognitive performance assessment into the analysis of autopsy procedures. The use of the Mackworth Clock Test allowed quantification of vigilance decrement associated with prolonged and demanding dissections. While both obese and control cases showed some degree of post-autopsy attentional decline, the magnitude of vigilance decrement was greater in obese cases, closely paralleling the longer procedural duration. The observed association between longer procedural duration and increased missed stimuli is consistent with previous human-factors research on fatigue during prolonged tasks.

The morphometric observation that subcutaneous adipose tissue thickness exceeded 4 cm in all cases with BMI ≥ 35 kg/m² provides an objective anatomical correlate to the observed procedural difficulties. Increased adipose thickness at the thoracoabdominal incision site translated into deeper incision planes, delayed access to body cavities, and prolonged dissection times, particularly during exposure and mobilization of deep or adipose-surrounded organs such as the liver, pancreas, adrenal glands, and pelvic organs.

It should be noted that cardiac examination protocols may vary across forensic institutions, particularly with regard to the timing of dissection in relation to fixation. While in many centers the heart is dissected fresh during the autopsy, alternative approaches involving delayed detailed examination after fixation are also described. Such variability may influence both procedural workflow and total autopsy duration, as fresh dissection requires additional time during the active autopsy phase, whereas deferred examination may redistribute workload to subsequent steps. In the present study, a standardized approach including fresh cardiac dissection was adopted for all cases in order to ensure methodological consistency. Nevertheless, inter-institutional differences in cardiac examination protocols should be considered when comparing autopsy duration across different settings.

The present findings support the relevance of workload-related human factors in forensic autopsy practice. However, the vigilance-related component of the study should be interpreted as exploratory and hypothesis-generating, since cognitive assessment was not designed to evaluate diagnostic performance or autopsy accuracy.

The study has limitations that warrant consideration. Although the inclusion of a matched control group enhances interpretability, the overall sample size remains moderate. The qualitative assessment of technical difficulty, while supported by consistent morphometric observations, remains partly subjective and could be further refined through future studies incorporating objective metrics of physical effort or motion analysis. The assessment of operator vigilance was conducted within a fixed autopsy team, which may limit generalizability. This design introduces a potential risk of pseudo-replication, limits external generalizability, and does not allow separation of individual operator characteristics from workload-related effects.

In conclusion, this study suggests that obesity is associated with longer and technically more demanding forensic autopsies under standardized conditions. Obese decedents require longer and more technically demanding examinations than non-obese controls, and increasing obesity severity is associated with progressive procedural prolongation and increased vigilance decrement scores in operators. Further investigations involving multiple operator teams and larger cohorts are required to better characterize workload-related cognitive effects in forensic autopsy practice.
